# Epigenetic Effects of Environmental Chemicals Bisphenol A and Phthalates

**DOI:** 10.3390/ijms130810143

**Published:** 2012-08-15

**Authors:** Sher Singh, Steven Shoei-Lung Li

**Affiliations:** 1 Department of Life Science, College of Science, National Taiwan Normal University, Taipei 116, Taiwan; E-Mail: sher@ntnu.edu.tw; 2 Graduate Institute of Clinical Medicine, Kaohsiung Medical University, Kaohsiung 807, Taiwan; 3 Center of Excellence for Environmental Medicine, Kaohsiung Medical University, Kaohsiung 807, Taiwan

**Keywords:** bisphenol A, phthalates, epigenetics, toxicogenomics, diseases

## Abstract

The epigenetic effects on DNA methylation, histone modification, and expression of non-coding RNAs (including microRNAs) of environmental chemicals such as bisphenol A (BPA) and phthalates have expanded our understanding of the etiology of human complex diseases such as cancers and diabetes. Multiple lines of evidence from *in vitro* and *in vivo* models have established that epigenetic modifications caused by *in utero* exposure to environmental toxicants can induce alterations in gene expression that may persist throughout life. Epigenetics is an important mechanism in the ability of environmental chemicals to influence health and disease, and BPA and phthalates are epigenetically toxic. The epigenetic effect of BPA was clearly demonstrated in viable yellow mice by decreasing CpG methylation upstream of the Agouti gene, and the hypomethylating effect of BPA was prevented by maternal dietary supplementation with a methyl donor like folic acid or the phytoestrogen genistein. Histone H3 was found to be trimethylated at lysine 27 by BPA effect on EZH2 in a human breast cancer cell line and mice. BPA exposure of human placental cell lines has been shown to alter microRNA expression levels, and specifically, miR-146a was strongly induced by BPA treatment. In human breast cancer MCF7 cells, treatment with the phthalate BBP led to demethylation of estrogen receptor (ESR1) promoter-associated CpG islands, indicating that altered ESR1 mRNA expression by BBP is due to aberrant DNA methylation. Maternal exposure to phthalate DEHP was also shown to increase DNA methylation and expression levels of DNA methyltransferases in mouse testis. Further, some epigenetic effects of BPA and phthalates in female rats were found to be transgenerational. Finally, the available new technologies for global analysis of epigenetic alterations will provide insight into the extent and patterns of alterations between human normal and diseased tissues. *In vitro* models such as human embryonic stem cells may be extremely useful in bettering the understanding of epigenetic effects on human development, health and disease, because the formation of embryoid bodies *in vitro* is very similar to the early stage of embryogenesis.

## 1. Introduction

Plastics are widely used in modern life, and their unbound chemicals bisphenol A (BPA) and phthalates can leach out into the surrounding environment. BPA and phthalates have recently attracted the special attention of the scientific community, regulatory agencies and the general public because of their high production volume, widespread use of plastics, and adverse health effects [1]. BPA is now used in the production of polycarbonate plastic containers such as baby bottles and epoxy resins that line metal cans for food and beverages. BPA is also used as a plasticizer to soften and increase the flexibility of polyvinyl chloride (PVC) plastic products. BPA has another medical use in dental sealants and composites used for filling. It is thought that human exposure mainly occurs through food and drink. However, exposure may also occur through dermal contact with thermal paper, used widely in cash register receipts. Phthalates are a group of similar diesters of phthalic acid used as plasticizers to soften and increase the flexibility of PVC plastics [2]. Human exposure to phthalates mainly occurs through foods, because of their uses in wrapping materials and food processing [3]. When ingested through food contamination, diethylhexyl phthalate (DEHP) is converted by intestinal lipases to mono-(2-ethylhexyl) phthalate (MEHP), which is then preferentially absorbed. Dibutyl phthalate (DBP) is used as a component of latex adhesives. It is also used in cosmetics and other personal care products, as a plasticizer in cellulose plastics, and as a solvent for dyes [4]. Monobutyl phthalate (MBP) is the toxic metabolite of DBP and butylbenzyl phthalate (BBP).

Epigenetics is the study of heritable changes in gene expression occurring without changes in DNA sequence. Epigenetic mechanisms include DNA methylation, histone modifications (acetylation, methylation, phosphorylation, ubiquitination, sumoylation and ADP ribosylation), and expression of non-coding RNAs (including microRNAs). In mammals, DNA methylation patterns are established during embryogenesis through the coopearation of DNA methyltransferases (DNMTs) and associated proteins. DNMT1 is responsible for the maintenance of methylation patterns throughout DNA replication (*i.e.*, specific for hemi-methylated sequences). DNMT2 may be involved in embryonic stem cells and potential RNA methylation. DNMT3A and DNMT3B are involved in active *de novo* DNA methylation at CpG sites. The early developmental period is thought to be the most susceptible to epigenetic insults because the DNA synthesis rate is high, and the elaborate DNA methylation patterning and chromatin organization required for normal tissue development is established at this time [5].

Epigenetics can influence the gene expression profiles of most organs and cell types. Furthermore, epigenetics is an important mechanism in the ability of environmental chemicals to influence human health and disease [6]. Environmental chemicals such as BPA and phthalates may play some critical roles in the etiology of many human disease risks [5,7,8]. Multiple lines of evidences from *in vitro* and *in vivo* models have established that epigenetic modifications caused by *in utero* exposure to environmental toxicants can induce alterations in gene expression that may persist throughout life. Thus, the environmentally induced epigenetic changes become increasingly relevant to human health and disease [9–11].

In the last few years, many investigations have examined the relationships between exposure to environmental chemicals and epigenetic effects, and identified several toxicants that modify epigenetic marks. Most of these studies conducted so far have focused on DNA methylation, whereas only a few recent investigations have studied the effects of environmental chemicals on histone modifications and expression of microRNAs [12]. Here, we review the epigenetic effects, as well as toxicogenomics, toxicities and health effects, of environmental toxicants BPA and phthalates derived from *in vitro* models, animal and human studies.

## 2. Toxicities and Health Effects of Bisphenol A and Phthalates

BPA and phthalates have long been known to have weak estrogenic properties and act as endocrine-disruptors owing to their ability to compete with endogenous steroid hormones binding to receptors. BPA was originally discovered as an artificial estrogen, and its estrogenic effect was used to enhance the rapid growth of cattle and poultry. BPA was also used for a few years as estrogen replacement for women. Since BPA can bind weakly to estrogen receptors ESR1 and ESR2, it is likely to be an endocrine disruptor. The impacts of BPA exposure on human health has been extensively reviewed and reported by the National Toxicology Program-Center for the Evaluation of Risks to Human Reproduction [13]. There is extensive literature showing the adverse effects of acute exposure of low doses of BPA in experimental animals [14,15]. Epidemiological studies had found associations between blood levels of BPA in women and impaired health, including endometrial hyperplasia and obesity [16]. BPA had been shown to have adverse health effects, including secondary sexual developmental changes and neurobehavioral alterations, in fetal through early childhood development [17]. Elevated exposure of pregnant women and children is of particular concern because of known windows of vulnerability to BPA that put the developing fetus and children at higher risk, compared with adults exposed to the same levels of BPA [14,18].

The impacts of phthalate exposure on human health have also been extensively reviewed and reported by the National Toxicology Program-Center for the Evaluation of Risks to Human Reproduction [19]. There is sufficient evidence in rodents that phthalate exposure causes developmental and reproductive toxicities. In humans, dysmorphic disorders of the genital tract, observed in male infants, were significantly associated with prenatal exposure to phthalates [20]. DBP/BBP/MBP were shown to have profound effects on the male reproductive development if exposure occurred during the critical periods of sexual differentiation (*i.e.*, late in the gestation). The phenotypic alterations observed in male offspring rats exposed to DBP/BBP/MBP during the perinatal period had remarkable similarities with common human reproductive disorders, including cryptorchidism, hypospadias and low sperm counts [21]. The antiandrogenic activities of phthalate mixtures and bisphenol A display additive interactions. They show a tendency to synergistic activities at high and antagonistic activities at low concentrations [22].

Biomonitoring of BPA through human blood and/or urine testing may underestimate the total body burden of this potential toxicant. Sweat analysis should be considered as an additional method for monitoring bioaccumulation of BPA in humans. Induced sweating appears to be a potential method for elimination of BPA [23].

## 3. Toxicogenomics of Bisphenol A and Phthalates

In the Comparative Toxicogenomics Database [24], BPA and the five most frequently curated phthalates (DEHP/MEHP and DBP/BBP/MBP) were found to have 1232 and 265 interactions with unique genes/proteins, respectively [25,26]. The GeneGo pathway maps, GeneGo processes, GeneGo toxicity networks and GeneGo diseases of the 1232 unique genes/proteins interacting with BPA were compared using MetaCore with those of the 265 unique genes/proteins interacting with five phthalates. BPA and phthalates were found to exhibit similar toxicogenomics, as well as adverse effects on human health, owing to their 89 common interacting genes/proteins. All of the top ten GeneGo pathway maps with highest probabilities were from the 89 common genes/proteins interacting with both BPA and phthalates, while those interacting with either BPA- or phthalate-specific genes/proteins had lower and little probabilities. All top 10 BPA- and phthalate-specific GeneGo processes were similar to those of the 89 common genes/proteins. It is of importance that five of the top 10 GeneGo toxicity networks predicted by the 89 common genes/proteins were involved in inflammation, because many chronic human diseases are due to immune and inflammatory dysfunctions [27]. It is also of interest that six of the top 10 GeneGo diseases were urogenital, prostatic, male genital, female genital, endometrial, and breast neoplasms. The diseases and disorders, as well as molecular and cellular functions, and physiological system development and functions, of the 89 common genes/proteins interacting with both BPA and phthalates were further analyzed using IPA, and cancer, developmental disorder and reproductive diseases were found to be the top three categories. Finally, these 89 genes/proteins may serve as biomarkers to assay the toxicities of environmental chemicals BPA and phthalates leached out from the widely used plastics.

## 4. Epigenetic Effects of Bisphenol A and Phthalates

**Figure 1:**
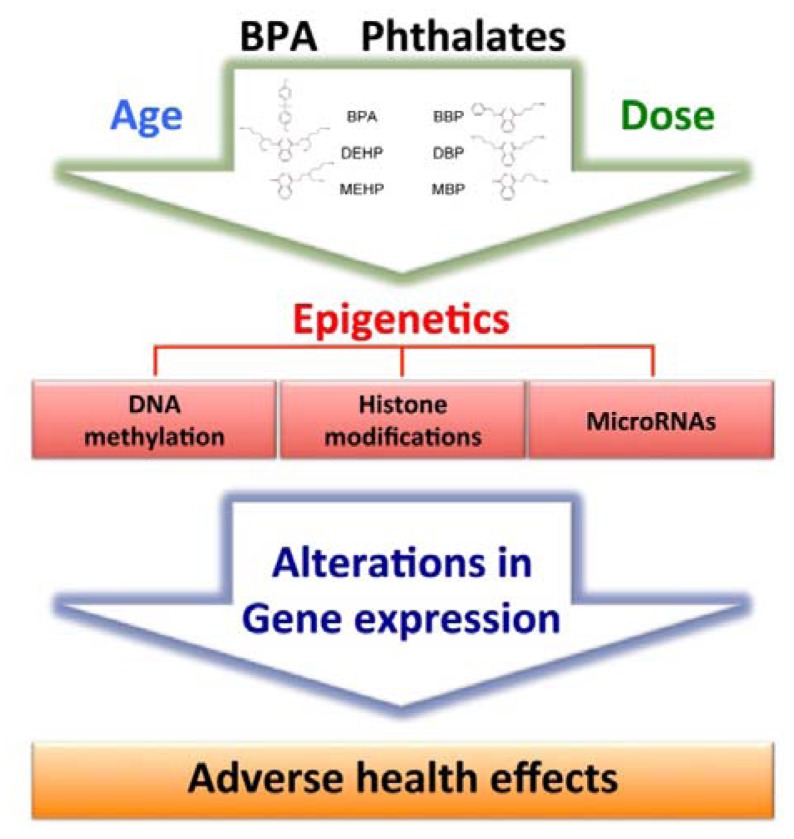
Epigenetic mechanisms of bisphenol A and phthalates.

Bisphenol A (BPA) and phthalates (DEHP/MEHP and DBP/BBP/MBP) are epigenetically toxic (Figure 1 and Table 1). The epigenetic effect of BPA was clearly demonstrated in viable yellow mice [28]. The maternal exposure to BPA shifted the coat color distribution of viable yellow mouse offspring toward yellow by decreasing CpG methylation in the IAP retrotransposable sequence inserted upstream of the Agouti gene. Interestingly, this effect on DNA methylation and the associated change in coat color of the exposed animals were prevented by maternal dietary supplementation with a source of methyl group such as folic acid or the phytoestrogen genistein [29].

The *in utero* and neonatal exposure to low doses of bisphenol A (BPA) and/or phthalates (DEHP/MEHP and BBP/DBP/MBP) may cause DNA hypermethylation/hypomethylation at CpG islands near gene promoter regions, histone modifications (acetylation, methylation, phosphorylation, ubiquitination, sumoylation and ADP ribosylation), and expression of non-coding RNAs, including microRNAs. These epigenetic marks can induce up/down alterations in gene expression that may persist throughout a lifetime. These permanent changes will result in adverse health effects such as neural and immune disorders, infertility, and late-onset complex diseases (cancers and diabetes). The transient exposure to BPA and phthalates of gestating female rats was further shown to be a transgenerationally differential DNA methylation of the F3 generation.

Exposure to endocrine disrupting chemicals such as BPA and phthalates is of particular concern in the context of development. Neonatal exposure of rats to BPA resulted in an increased incidence of prostate intraepithelial neoplasia, and the prostate tissues showed consistent methylation changes. For example, the phosphodiesterase type 4 variant 4 (Pde4d4) gene of the rat was found to have hypomethylation in the regulatory CpG island and an elevated expression in the adult prostate [30,31]. Neonatal exposure of the rat to BPA was also reported to alter the promoter methylation and expression of nucleosome binding protein-1 (Nsbp1) and hippocalcin-like 1 (Hpcal1) genes [32]. The neonatal exposure to BPA was shown to induce hypermethylation of estrogen receptor promoter regions in rat testis, indicating methylation mediated epigenetic changes as one of the possible mechanisms of BPA induced adverse effects on spermatogenesis and fertility [33].

BPA has been shown to alter the methylation status of the *Hoxa10* gene in mouse *in utero* exposure model [34]. The *in utero* BPA treatment increased the expression of the developmental homeobox gene *Hoxa10* in the uterus of female offspring at two weeks of age. This change in gene expression was associated with significant demethylation of specific CpG sites in both promoter and intron of the *Hoxa10* gene. Genome-wide effects of BPA on DNA methylation in brain tissue have also been investigated. Maternal exposure to BPA was associated with either hypo- or hyper-methylation of the promoter-associated CpG islands in several loci in the fetal mouse brain [35]. Gene-specific changes were confirmed at 13 loci, and changes in DNA methylation state of two genes, encoding transport-related proteins, were associated with altered gene expression profiles. Exposure of human primary breast epithelial cells to low-dose BPA was reported to increase DNA methylation at CpG islands of lysosomal-associated membrane protein 3 (*LAMP3*) gene and repress the expression of *LAMP3* gene [36].

BPA effects on histone modifications were found to increase expression of the histone methyltransferase Enhancer of Zeste Homolog 2 (*EZH2*) level in human breast cancer MCF7 cells and mammary glands of six-week-old mice exposed to BPA *in utero* [37]. Both *in intro* and *in vivo*, these changes were accomplished by an increase in histone H3 trimethylation at lysine 27, which is the main histone modification catalyzed by EZH2 and is typically associated with gene expression [38].

Concerning microRNAs (miRNAs), BPA exposure of human placental cell lines has been shown to alter miRNA expression levels, and specifically, miR-146a was strongly induced by BPA treatment. This resulted in both slower proliferation rate and higher sensitivity to the DNA damaging agent bleomycin [39]. A mouse sertoli cell line TM4 exposed to BPA for 24 h was reported to have two-fold up or down-regulated 37 miRNAs, and most of miRNAs were down-regulated over the course of BPA treatment [40].

As to phthalates, treatment of human breast cancer MCF7 cells with BBP led to the demethylation of estrogen receptor (ESR1) promoter-associated CpG islands, indicating that altered *ESR1* mRNA expression by BBP is related to aberrant DNA methylation in the promoter region of the receptor gene [41]. Maternal exposure to DEHP was shown to increase DNA methylation and expression levels of DNA methyltransferases in mouse testis. Fetal testis was a main target for DEHP as evidenced in testicular dysgenesis syndrome due to a reduction in insulin-like hormone 3 (*INSL3*) expression and testosterone production [42].

Molecular mechanisms that underlie the long-lasting effects of BPA and phthalates continue to be elucidated, and they likely involve disruption of epigenetic programming of gene expression during development. It will be important to determine whether epigenetic markers in more accessible tissues correlate with epigenetic markers in target tissues. Many studies strongly imply that exposures to endocrine-disrupting chemicals (EDCs) may have cumulative adverse effects on future generations, and that these effects could be mediated through epigenetic mechanisms [43].

Finally, the transient exposure to a plastic mixture (BPA and phthalates) of gestating female rats during the period of embryonic sex determination was shown to promote early-onset female puberty transgenerationally (F3 generation) and decrease the pool size of ovarian primordial follicles. Spermatogenic cell apoptosis was also affected transgenerationally, and differential DNA methylation of the F3 generation sperm promoter regions was found in all exposed lineage males [44].

## 5. Conclusion and Remarks

The hypomethylation of the mouse Agouti gene caused by exposure to BPA can be prevented by maternal dietary supplementation with a source of methyl group [29]. However, it remains to be investigated if any bioaccumulation of epigenetic impacts can be reversed/eliminated after exposure to BPA and phthalates is discontinued. The differential DNA methylation was reported to be transgenerational after exposure of gestating female rats to mixture of BPA and phthalate, but the synergistic impact of both BPA and phthalate remain to be determined.

The growing evidence indicates that epigenetics holds substantial potential for developing biological markers to predict which chemicals would put exposed subjects at risk and which individuals would be more susceptible to developing disease. It is still important to note that the mechanisms by which environmental toxicants modulate the epigenetic landscape of individual cells are yet to be elucidated in order to better understand the biology of epigenetic alterations and the health effects of toxic exposures on these disease-associated epigenetic alterations. Better defined mechanisms will lead to better prediction of the toxic potential of environmental chemicals such as BPA and phthalates and allow for more targeted and appropriate disease prevention strategies.

In human studies, the use of laboratory methods with enhanced precision, sensitivity and coverage will be required, so that epigenetic changes can be detected as early as possible and well ahead of disease diagnosis. New technologies available now allow for global analysis of epigenetic alterations and these may provide insight into the extent and patterns of alterations between human normal and diseased tissues. Appropriate *in vitro* models must be considered. In this context, human embryonic stem cells may be extremely useful in bettering the understanding of epigenetic effects on human development, health and disease, because the formation of embryoid bodies *in vitro* is very similar to the early stage of embryogenesis [45,46].

## Figures and Tables

**Table 1:** Epigenetic effects of bisphenol A (BPA) and phthalates.

Chemicals	Epignetic alterations	Genes	Organisms	References
BPA	DNA hypomethylation	Agouti	Mouse	[28,29]
BPA	DNA hypomethylation	Pde4d4	Rat	[30,31]
BPA	DNA hypomethylation	Nsbp1	Rat	[32]
	DNA hypermethylation	Hpcal1		
BPA	DNA hypermethylaton	ESR1&2	Rat	[33]
BPA	DNA hypomethylation	Hoxa10	Mouse	[34]
BPA	DNA hypomethylation or hypermethylation	13 genes	Mouse	[35]
BPA	DNA hypermethylation	LAMP3	Human	[36]
BPA	Histone modification	H3K27me3	Human Mouse	[37,38]
BPA	Induction of microRNA	miR-146a	Human	[39]
BPA	Up/down-regulated miRNAs	37 miRNAs	Mouse	[40]
Phthalate BBP	DNA hypomethylation	ESR1	Human	[41]
Phthalate DEHP	DNA hypermethylation	INSL3	Mouse	[42]
Mixture of BPA & phthalates	Differential DNA methylation Transgeneration (F3)		Rat	[43]

## Abbreviations

BBP: butylbenzyl phthalate
BPA: bisphenol A
DBP: dibutyl phthalate
DEHP: diethylhexyl phthalate
EDCs: endocrine-disrupting chemicals
IPA: Ingenuity Pathways Analysis
MBP: monobutyl phthalate
MEHP: mono-(2-ethylhexyl)phthalate
PVC: polyvinyl chloride
